# Automatic Detection Method for Cancer Cell Nucleus Image Based on Deep-Learning Analysis and Color Layer Signature Analysis Algorithm

**DOI:** 10.3390/s20164409

**Published:** 2020-08-07

**Authors:** Hsing-Hao Su, Hung-Wei Pan, Chuan-Pin Lu, Jyun-Jie Chuang, Tsan Yang

**Affiliations:** 1 Department of Otorhinolaryngology-Head and Neck Surgery, Kaohsiung Veterans General Hospital, Kaohsiung 81362, Taiwan; shsu@vghks.gov.tw; 2 Department of Pharmacy and Graduate Institute of Pharmaceutical Technology, Tajen University, Pingtung 90741, Taiwan; 3 School of Medicine for International Students, College of Medicine, I-Shou University, Kaohsiung 84001, Taiwan; hwpan@isu.edu.tw; 4 Department of Information Technology, Meiho University, Pingtung 91202, Taiwan; d40614139@go.meiho.edu.tw; 5 Department of Health Business Administration, Meiho University, Pingtung 91202, Taiwan; x00002115@meiho.edu.tw

**Keywords:** nucleus, mitosis, micronuclei, artificial intelligence, computer vision, convolutional neural network

## Abstract

Exploring strategies to treat cancer has always been an aim of medical researchers. One of the available strategies is to use targeted therapy drugs to make the chromosomes in cancer cells unstable such that cell death can be induced, and the elimination of highly proliferative cancer cells can be achieved. Studies have reported that the mitotic defects and micronuclei in cancer cells can be used as biomarkers to evaluate the instability of the chromosomes. Researchers use these two biomarkers to assess the effects of drugs on eliminating cancer cells. However, manual work is required to count the number of cells exhibiting mitotic defects and micronuclei either directly from the viewing window of a microscope or from an image, which is tedious and creates errors. Therefore, this study aims to detect cells with mitotic defects and micronuclei by applying an approach that can automatically count the targets. This approach integrates the application of a convolutional neural network for normal cell identification and the proposed color layer signature analysis (CLSA) to spot cells with mitotic defects and micronuclei. This approach provides a method for researchers to detect colon cancer cells in an accurate and time-efficient manner, thereby decreasing errors and the processing time. The following sections will illustrate the methodology and workflow design of this study, as well as explain the practicality of the experimental comparisons and the results that were used to validate the practicality of this algorithm.

## 1. Introduction

A dysregulated cell cycle is a common phenomenon in human cancers, and many therapeutic strategies focus on inhibiting the proliferation of cancerous cells. Generally, a disorder in the mechanism regulating chromosome segregation in cancer cells causes the cell cycle to become dysregulated along with the overexpression of mitosis-regulating factors, resulting in carcinogenesis. In most situations, this behavior is attributed to the dysregulation of chromosome segregation in cancer cells [1]. However, different cancer cells have different levels of chromosome changes (either losses or gains). This phenomenon is called instability and may result from molecular changes caused by chromosome segregation during mitosis [1,2]. Centrosome defects during mitosis can lead to chromosome mis-segregation and aneuploidy, resulting in genome instability and, more importantly, are the primary driving force for malignant transformation and tumor progression [3,4]. Multiple studies have confirmed that chromosome instability influences cancer cells in two different ways. Mild chromosome instability in cancer cells will slightly promote growth and thereby facilitate malignancy; however, high chromosome instability will lead to cell death and is a mechanism that inhibits tumor growth [5]. Recently, a new strategy was developed to combat cancer cells using the phenomenon of chromosome instability and aneuploidy [6]. Therefore, many studies and reviews have reported that it is possible to eliminate highly proliferative cancer cells specifically by practicing cell death induction by producing chromosomes in cancer cells that are more unstable and inducing aneuploidy during mitosis [7,8,9]. There are two causes that make chromosomes unstable: errors in the process of the spindle fiber assembly and chromosome segregation during mitosis. Therefore, if there is a specific molecule that can disrupt the process of spindle fiber assembly or chromosome segregation during mitosis, a more unstable chromosome status can be induced, which can be developed into a new treatment strategy.

The level of genome instability can be measured in several ways, including flow cytometry, fluorescence in situ hybridization, comparative genomic hybridization, and allele typing. Random amplified polymorphic DNA is a polymerase chain reaction-based fingerprinting technique in which short arbitrary sequence primers are used to amplify random DNA fragments. Most cancer cells are aneuploid and contain an abnormal number of chromosomes, primarily because of their increased chromosome mis-segregation rate [10,11]. One factor worth noting is the presence of cell nuclei exhibiting micronuclei, which are attributed to a cytoplasmic chromatin mass that has the appearance of a small nucleus. These cell nuclei are attributed to anaphase lag or centromere fragments [12]; moreover, mitotic defects and genome instability are present. Therefore, mitotic defects and micronuclei can be used as biomarkers for chromosome instability. However, researchers often have to use microscopy or take multiple images for the manual enumeration of cell nuclei with mitotic defects and micronuclei. This is extremely tedious and usually generates numerous errors as DAPI (4′, 6-diamidino-2-phenylindole), a fluorescent dye, is used to stain the cell nucleus, and Rhodamine Phalloidin (R415) is used to stain the cytoskeleton during the inspection. However, not every cell can be stained evenly (each sample contains ~20,000 cells). Therefore, an automatic image interpretation method could assist laboratory staff in analysis, increase efficiency and accuracy, and reduce human error. Computer vision has rapidly developed and is now used in many fields. Tasks that used to be performed by human observation can now be accomplished by computer vision.

Computer vision has several advantages, such as stability, standardization, long-term operation, and consistency. For example, Sharma et al. used deep learning to perform cell nuclei directional segmentation and gastric cancer cell classification from heterogeneous histopathology images [13]. Cell image segmentation was then performed using binary threshold techniques. However, the quality of the results from the binary threshold technique can be negatively influenced by several factors, such as noise and uneven backgrounds in the images or images with an obvious cell texture. De Sousa proposed a segmentation method for the curvature analysis of merged blocks, while the Otsu threshold is used to separate blocks [14]. Kowal et al. proposed a multilabel calculation method to obtain blocks and used this method to search for cell nuclei morphology [15]. Finally, from microscopy images, texture, and topological features are used to determine whether tumors are benign or malignant. Song et al. proposed using contour fragments produced from cell blocks to plot graphs of the minimal energy function, and fragments from the same cytoplasm were placed in the same set to achieve automatic segmentation of the overlapping cytoplasm [16]. Vununu et al. proposed a deep feature extraction method for HEp-2 cell image classification [17]. Kucharski et al. proposed a semi-supervised segmentation method to solve the problem of ground-truth images segmentation for the detection of nests of nevus cells in histopathological images of skin specimens [18]. The aforementioned studies did not focus on micronucleus detection. Ramadhani and Purnami (2013) used CellProfiler for the automatic analysis of cell images of binucleated cells and micronuclei [19]. The authors compared the detected cell numbers of the binucleated cells and micronuclei that were obtained using manual and CellProfiler counting. CellProfiler only detects isolated micronuclei (unconnected cell nuclei). However, micronuclei are generally located at the edges of the cell nuclei and are connected to the nucleus. Moreover, the software uses the thresholding algorithm to remove backgrounds (such as Otsu or minimum cross-entropy thresholding methods). Thresholding easily distinguishes the cell nuclei with a lower brightness and regions with nonapparent micronuclei as the backgrounds. It is also easy to generate many small fragments and identify them as micronuclei. To solve the excessive time consumption and errors of manual work, as well as detect the micronuclei connected to the nucleus, this study adopts an automated technique based on artificial intelligence and computer vision to spot colon cancer cells with genome instability. This method uses the convolutional neural networks (CNN) [20] technique to identify normal cell nuclei, and we propose a novel color layer signature analysis (CLSA) algorithm to detect cells with mitotic defects and micronuclei. By implementing this new approach, researchers can detect cancer cells more accurately and quickly; furthermore, manually induced errors and processing time can both be reduced. 

In recent years, artificial intelligence based on neural networks has seen significant breakthroughs, particularly in the area of image recognition. Deep-learning neural networks can be used for the image recognition of many objects; even if the target objects have an angular offset, are rotated, or remain incomplete, programs can still recognize them. One of the most common neural networks is CNN, which can be used to input training data for stratified learning and does not require setting feature values for recognition. In this past, this solved the problem of identifying valid features for image recognition. Different strata of neural networks are used to mimic human learning to capture different structures in images. Because of its different tasks, CNN requires a large volume of samples with known data for training to obtain the required weighting parameters. Faster and more precise neural network algorithms can be developed using CNN as the core framework. Examples include AlexNet (2012) [21], OverFeat (2013) [22], RCNN (Regions with CNN features) (2014) [23], ZFNet (2014) [24], GoogleNet (2015) [25], SPPNets (Spatial Pyramid Pooling in Deep Convolutional Networks) (2015) [26], Fast R-CNN (2015) [27], VGGNet (2015) [28], SSD (Single Shot Multibox Detector) (2016) [29], ResNet (Deep Residual Learning for Image Recognition) (2016) [30], Mask R-CNN (2016) [31], Faster R-CNN (2017) [32], DenseNet (Densely Connected Convolutional Networks) (2017) [33], and YOLO (You Only Look Once) (2016–2018) [34,35,36]. The use of CNN for cell image recognition (localization) is better than conventional image processing methods (such as using binary images for regional segmentation and localization). As the cell nucleus is a small image object and the YOLO algorithm [36] offers good recognition results for small object images, we used the YOLO algorithm (version 3) for recognition to analyze colon cancer cells more accurately. 

In this study, we train YOLO using samples featuring only normal cells. In other words, cells with cell nuclei exhibiting clear micronuclei, with mitotic defects excluded from the training data. In this case, cells not recognized will then be considered as having cell nuclei with mitotic defects or micronuclei. Given that certain cell nuclei with micronuclei cannot be easily spotted, we introduce the CLSA. In CLSA, a contour line geometric shape analysis is performed for every color layer after color quantization. This method can identify micronuclei with a low brightness, and changes in the contour lines of various layers are used as the basis for micronucleus detection. Therefore, in this study, YOLO is used to distinguish normal cell nuclei from cells with mitotic defects or apparent micronuclei in their nuclei. For the next step, CLSA is used to identify cell nuclei with micronuclei in the normal cell nuclei. The number of cell nuclei with mitotic defects and micronuclei is then used as a reference for targeted tumor inhibition, i.e., the greater the number of cancer cells with high chromosome instability, the better the effects that can be obtained from tumor inhibition. This study will also elaborate on the methodology, experimental comparisons, and results that are used to validate the practicality of this algorithm.

## 2. Materials and Methods 

**Figure 1:**
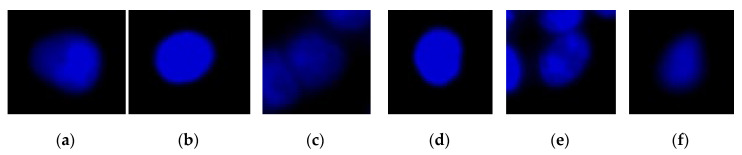
Images of the normal cell nucleus: (**a**) type 1; (**b**) type 2; (**c**) type 3; (**d**) type 4; (**e**) type 5; (**f**) type 6.

**Figure 2:**
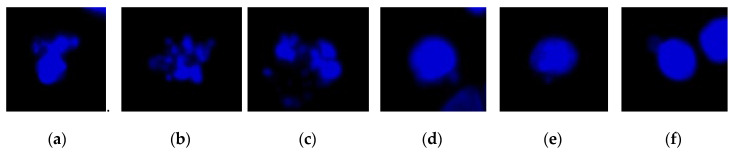
Cell nuclei images: (**a** **c**) image of the cell nucleus with mitotic defects; (**d** **f**) image of the cell nucleus with the micronucleus.

**Figure 3:**
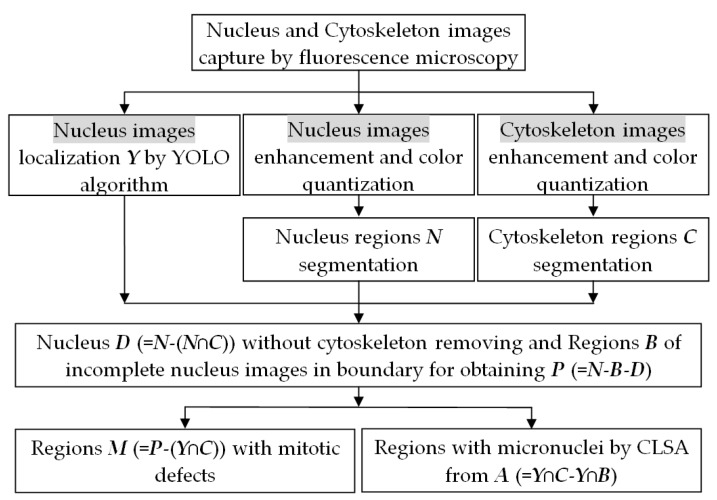
The processing procedure of the proposed method for the automatic cell detection of nuclei from a colon cancer image.

To analyze colon cancer cell images, we propose an automatic method for cell detection based on the results of deep-learning image analysis and combine this method with the CLSA algorithm for the additional detection of cell nuclei with mitotic defects and micronuclei. The result providing the numbers and ratios of these cell nuclei can be used as a reference for determining the efficacy of targeted therapy drugs. For this method, we first obtained cell nucleus images by fluorescence microscopy. Subsequently, YOLO was used to distinguish normal cell nuclei (Figure 1) from cell nuclei with mitotic defects (Figure 2a–c), as well as from cell nuclei with clear micronuclei (Figure 2d–f). Subsequently, CLSA was used on normal cell nuclei to detect cell nuclei with micronuclei. Figure 3 shows the processing procedure of the proposed automatic image detection method, which is divided into three parts: YOLO is used to recognize (locate) normal cell nuclei in the raw images (defined as ***Y***), and the raw nucleus images and raw cytoskeleton images are used for color quantization. Subsequently, the background regions are removed after image quantization, while the nucleus regions (defined as ***N)*** and cytoskeleton regions (defined as ***C***) are retained. Some of the cell nuclei that do not overlap with the cytoskeleton are not included in the quantity statistics, which are called invalid regions. Subsequently, the incomplete nucleus regions exhibiting rough edges (defined as ***B***) are removed, and the regions without a cytoskeleton (defined as ***D*** (=***N***−(***N***∩***C***))) are also removed (∩: intersection operation). After removing regions ***B*** and ***D***, only the cell nucleus regions with mitotic defects and micronuclei remain (defined as ***P*** (=***N***−***B***−***D***)). The regions with mitotic defects (defined as ***M***) are determined by ***P***−(***Y***∩***C***). For the cell nuclei with micronuclei, CLSA is introduced to analyze the regions ***A*** (=***Y***∩***C***−***Y***∩***B***). The approaches used for image processing include image enhancement, color quantization, set operations, labeling, nucleus recognition, and CLSA analysis. The flowchart of a portion of the entire process is shown below.

### 2.1. Nucleus Image Recognition (Localization) by the YOLO Algorithm

Cell division is the foundation of cell proliferation. To obtain an accurate cell nuclei count, we used YOLO (version 3), developed by Redmon and Farhadi, to recognize and spot the cell nuclei in the images. The reason for using the YOLO algorithm is that YOLO provides good recognition results for small-sized cell nucleus images [36]. YOLO is based on DarkNet-53 as its core network and is an application of the multibox method. Unlike other algorithms, this method can be used to obtain greater image recognition, a higher mean average precision, and a shorter calculation time, which meets the calculation requirements. YOLO uses the multibox candidate region selection method for image object recognition. Moreover, the softmax, which is parallel to the box regressor and box classifier, is added such that the resolutions in the candidate region in multibox can be repurposed for object recognition. In this manner, the category of the objects can be determined without further processing. To improve the performance of the algorithm in processing small-sized objects, fine-grained features are added to YOLO. This is achieved by adding a passthrough layer in which the features in the superficial layer are connected to the deep layer. During processing, YOLO divides the images used for recognition or training into H × H grid cells, which are used to replace the ground truth box dataset to obtain the matching network’s prior box. Moreover, these grids are compatible with box matching in which the center of the object image being detected is inserted into the grid, which must identify the exact position and category of the object image. For feature capturing, YOLO uses the DarkNet-53 network, which is based on Darknet-19 and residual networks. DarkNet-53 is a deep-learning network that contains 53 feature extraction layers (including 52 convolutional layers and one connected layer). YOLO uses a 416 × 416 image for training and recognition and divides the image into 13 × 13 grid cells, which are used to replace the ground truth box dataset to obtain the matching network’s prior box. These grid cells are used for box matching in which the center of the object image being detected is inserted into the grid. The grid cell must identify the exact position and category of the object image in the grid. 

When training the networks, to obtain the best prediction results and detect the position of the object in the image, the mean squared error is designed for the loss function in YOLO [34]. The loss function includes calculating the errors of the bounding boxes’ coordinate regression, the source prediction, and the class score prediction. The loss function is shown in Equation (1) [34]. The symbol $1_{ij}^{\mathrm{obj}}$ is used to denote the *j*th bounding box of the *i*th grid to calculate a given object. In Equation (1), the first two terms indicate the bounding box coordinate regression of the position and the size error in the bounding box. The third and fourth terms are the calculations of the bounding box source prediction, and the last term is the calculation of the class score prediction.

(1) $$\begin{array}{c} \mathrm{Loss}\;\mathrm{Function}\;={\;\lambda }_{\mathrm{coord}}\overset{H^{2}}{\underset{i=0}{\sum }}\overset{B}{\underset{j=0}{\sum }}1_{ij}^{\mathrm{obj}}[{\left(x_{i}-{\hat{x}}_{i}\right)}^{2}+{\left(y_{i}-{\hat{y}}_{i}\right)}^{2}]\\ +\lambda_{\mathrm{coord}}\overset{H^{2}}{\underset{i=0}{\sum }}\overset{B}{\underset{j=0}{\sum }}1_{ij}^{\mathrm{obj}}\left[{\left(\sqrt{w_{i}}-\sqrt{{\hat{w}}_{i}}\right)}^{2}+{\left(\sqrt{h_{i}}-\sqrt{{\hat{h}}_{i}}\right)}^{2}\right]+\overset{H^{2}}{\underset{i=0}{\sum }}\overset{B}{\underset{j=0}{\sum }}1_{ij}^{\mathrm{obj}}{\left(C_{i}-{\hat{C}}_{i}\right)}^{2}\\ +\lambda_{\mathrm{noobj}}\overset{H^{2}}{\underset{i=0}{\sum }}\overset{B}{\underset{j=0}{\sum }}1_{ij}^{\mathrm{noobj}}{\left(C_{i}-{\hat{C}}_{i}\right)}^{2}+\overset{H^{2}}{\underset{i=0}{\sum }}1_{i}^{obj}\underset{c\in classes}{\sum }{\left(p_{i}\left(c\right)-{\hat{p}}_{i}\left(c\right)\right)}^{2} \end{array}$$

where $\lambda_{\mathrm{coord}}$, $\lambda_{\mathrm{noobj}}$ are constants; $x_{i},\;y_{i}$ are the center coordinates of the *i*th anchor box;$\;\;{\hat{x}}_{i},\;{\hat{y}}_{i}$ are the center coordinates of the *i*th known ground truth box; $\;w_{i},\;h_{i}$ are the width and height of the *i*th anchor box;$\;\;{\hat{w}}_{i},\;{\hat{h}}_{i}$ are the width and height of the *i*th ground truth box; $C_{i}$ is the confidence score of the *i*th objectness; $\;{\hat{C}}_{i}$ is the objectness of the *i*th ground truth box; $p_{i}\left(c\right)$ is the classification loss of the *i*th object; $\;{\hat{p}}_{i}\left(c\right)$ is the classification loss of the *i*th ground truth box; one input image is divided into an H×H grid; B is the number of bounding boxes predicted in each grid cell.

As there is no specific pattern for cell nuclei with mitotic defects, we excluded samples with cell nuclei exhibiting mitotic defects, as well as cell nuclei showing clear micronuclei, from the training data; only normal cell nuclei images were then used for training the networks. The bounding box is used for the image recognition results (defined as ***Y***). 

### 2.2. Image Color Quantization and Region Localization

During the preparation procedure for cell testing, laboratory staff stain cells to clearly observe the cells under a fluorescent microscope to obtain the cell count and distinguish cells with normal cell nuclei from those with mitotic defects or micronuclei. These data can then be used to assess the effects of drugs on inhibiting tumors. The level of even staining of the cells, the microscope magnification, the light intensity of the fluorescent microscope, and the cell texture in the images will affect the accuracy of the visual inspection of cells. Therefore, inspectors need an automatic image inspection method to assist them in this analysis, thereby reducing inspection errors and the need for repeated inspections. In the proposed automatic image detection method, to obtain clear cell images showing the regions and contours, images are first captured via fluorescence microscopy and then sharpened [37]. Subsequently, color quantization is used to obtain image regions and separate the cell nucleus region and background. The obtained cell nucleus region is then used for subsequent analyses. Common color quantization algorithms [37] include median cut, k-means clustering [38], and self-organizing maps. In this study, we use k-means clustering. Quantized color values determine the number of cell clusters. The greater the color value is, the greater the number of regions in the color layer are and the more calculations that are processed in subsequent regions. However, the lower the color value is, the fewer the regions and number of calculations that are processed. However, in the latter case, cell regions connect with neighboring regions more often, resulting in difficulty in dividing the cell nucleus regions. In this study, the quantized color value (defined as k) is defined as 10–20, which is sufficient for the detection goal. To focus on the initial regions to spot cells with mitotic defects and cells with abnormal nuclei, we set the threshold of the initial region at a level where the grayscale value is higher than the background (lowest value). 

### 2.3. Regions without Cytoskeleton Removal and Regions of Incomplete Nucleus Images in the Boundary

As most cell nuclei cannot be stained evenly, the cell nuclei in images cannot be maintained at the same level of brightness. To obtain every cell nucleus region, we inspected the contours of every color layer and use the region with the lowest quantized color value as the background, while the remaining region is considered to be the cell nucleus region (***N***). This method is better than binary processing and background removal, because it can retain cell nuclei with lower brightness and regions with nonapparent micronuclei. Because cell nuclei that do not overlap with the cytoskeleton (invalid regions) are not included in the enumeration, we performed color quantization for the cytoskeleton images, removed the background to obtain the cytoskeleton regions (***C***), and preserved the regions that overlap with the cytoskeleton to obtain the region of ***D*** (=***N***−(***N***∩***C***)). Region calculation and acquisition are performed using the labeling algorithm [37]. Moreover, it is necessary to remove the cell nuclei at the edges of images with incomplete nuclei (***B***). The final region retained is ***P*** (=***N***−***B***−***D***). These results are used for further processing to detect normal cell nuclei, cell nuclei with mitotic defects (***M*** (=***P***−(***Y***∩***C***)), and cell nuclei with micronuclei (***A*** (=***Y***∩***C***)). 

### 2.4. Color Layer Signature Analysis (CLSA) Algorithm 

**Figure 4:**
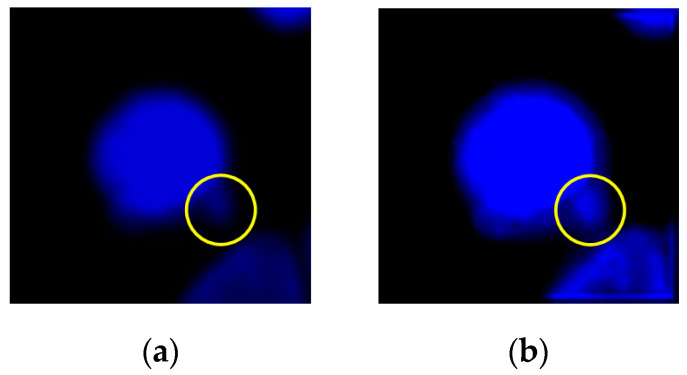
A nucleus with micronuclei (the yellow circle): (**a**) original cell image; (**b**) image enhancement for observation.

**Figure 5:**
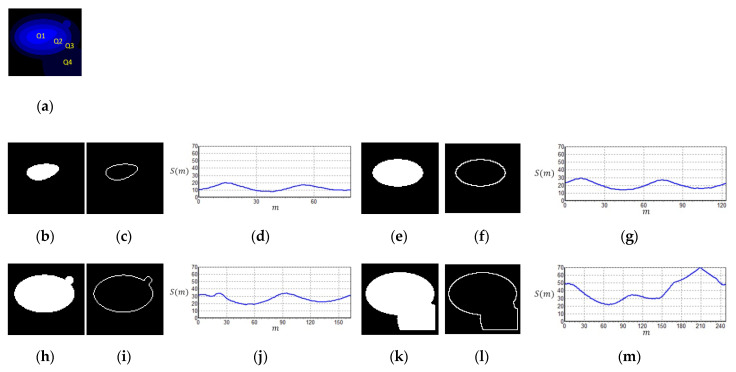
Signature of the color layer regions (S(m)): (**a**) four regions based on color quantization image; (**b**) Q1; (**c**) contour of Q1; (**d**) signature of Q1; (**e**) Q2; (**f**) contour of Q2; (**g**) signature of Q2; (**h**) Q3; (**i**) contour of Q3; (**j**) signature of Q3; (**k**) Q4; (**l**) contour of Q4; (**m**) signature of Q4.

**Figure 6:**
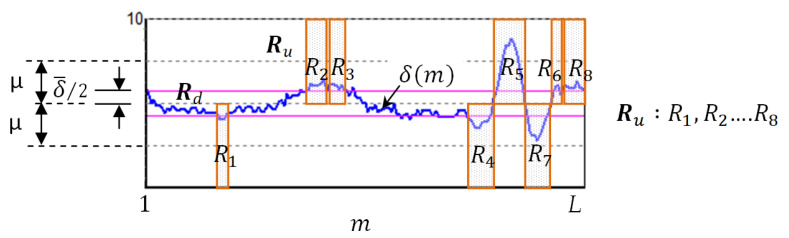
Short window energy curve and regions $R\;(=\{R_{u}\;,R_{d}\})$.

**Figure 7:**
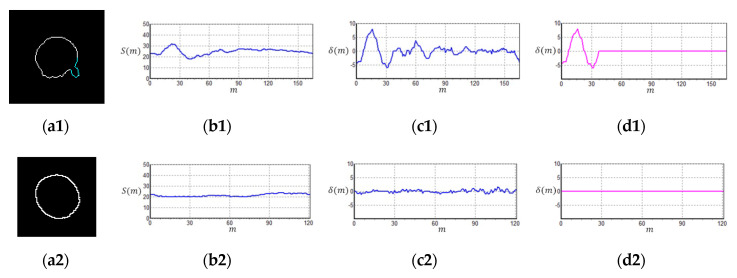
Micronucleus detection of real cell nuclei images: (**a1**,**a2**) contour of cell regions; (**b1**,**b2**) signature curves; (**c1**,**c2**) short window energy curve; (**d1**,**d2**) short window energy curves by screening sensitivity (μ = 4).

Micronuclei can be commonly found at the edges of cell nuclei (Figure 4), and certain micronuclei are not clearly visible. To make the micronuclei easier to spot, we enhanced the images shown in Figure 4a to obtain the results shown in Figure 4b, where the yellow circles represent the micronuclei. To detect cell nuclei with micronuclei, we propose a new algorithm known as CLSA. As micronuclei appear at the edges of cells where the contrast is low, they cannot be easily detected. For this purpose, the CLSA is used to analyze the cell region ***A*** (=***Y***∩***C***), which is recognized by YOLO. To show the changes in the signature curve of the cell nucleus with micronuclei in different color layers, we use manually plotted simulated cell nucleus images (Figure 5) to demonstrate the processing procedure in the CLSA. Figure 5 is used to simulate the regions obtained by thresholding with the four threshold values and shows a common cell image divided into four regions based on color quantization (Q1–Q4, Figure 5b,e,h,k). The lower the grayscale value is, the greater the areas are. The micronucleus regions are hidden in the color layer, as shown in Figure 5h. We converted the contour line (Figure 5i) in Figure 5h into a signature curve (Figure 5j). The effects of the micronucleus region on the signature curve are reported in Figure 5j. Based on this phenomenon, the CLSA algorithm detects the micronuclei in the contour lines of every layer. Figure 5b,e,h,k are regions showing cell nuclei with micronuclei. CLSA is then used to extract the contour lines in the region (the results in Figure 5c,f,i,l). Subsequently, Chaincode [37] is used to convert the distance between the center of the cell region and the contour line into a signature curve. After obtaining a signature curve (S(m), m is the variable of function S) (Figure 5d,g,j,m), the wavelet function (coefficients: $\left\{1/\sqrt{2},-1/\sqrt{2}\;\right\}$) in the wavelet Haar transform [37] is used for convolution to obtain the high-frequency information of the signature curve ( δ(m)). After obtaining the high-frequency wavelet signals, differentiation (ψ′(m)) is introduced to increase the differences between the signals. The data (circular padding) of 10 cumulative ψ′(m) values are used to show changes in the short window, which is called the short window energy curve (δ(m) (as in Equation (2)), where *L* is the data number of δ(m)). Note that the micronucleus appears as a mountain peak in δ(m). The mean ($\bar{\delta }$) in δ(m) (Equation (3)) is used to screen out the mountain peak R that is higher than $\bar{\delta }$/2 ( R = **{**$R_{u}\;,R_{d}$**},** $R_{u}$ is the region that exceeds $\bar{\delta }$/2). Equation (4) shows the region of $R_{d}$ in $\bar{\delta }$/2. The threshold value (known as sensitivity μ) is used to determine whether the peak (or valley) in the $R_{u}$ interval exceeds the range of μ. If a peak (or valley) that exceeds the μ range occurs in a few continuous layers, it is considered to be a micronucleus. If the peak (or valley) exceeds the μ range once, the peak may represent the texture of the cell nucleus. Considering Figure 6 as an example of the short window energy curve ( δ(m)), the region that exceeds $\bar{\delta }$/2 is $R_{u}$ ($R_{1},R_{2},\;\ldots ,R_{8}$)**,** and the region that exceeds the range of sensitivity μ is $R_{5}$, which continuously appears in more than three layers. This cell nucleus is determined to contain a micronucleus. The contour in Figure 7a1 was extracted from Figure 4; Figure 7a1 shows the cell nuclei with micronuclei, while Figure 7a2 does not contain a micronucleus. Figure 7b1,b2 are the signature curves of the cell nucleus region, and Figure 7c1,c2 are the short window energy curves. The sensitivity μ(=4) results in Figure 7c1,c2 are shown in Figure 7d1,d2. The area that exceeds the interval of sensitivity μ is roughly localized to the position of the micronucleus on the contour (cyan lines) (see Figure 7a1,a2). By screening sensitivity μ, the results in Figure 7d1 were obtained (Figure 7d2 shows the results of the control).

(2) $$\delta \left(m\right)=\sum_{t=1}^{t=10}\psi \prime \left(m+t\right),\;\psi \prime \left(m\right)=\psi \left(m+1\right)-\psi \left(m\right),\;\;m=1,2,3,\ldots ,\;L$$

(3) $$\bar{\delta }=\frac{1}{L}\overset{m=L}{\underset{m=1}{\sum }}\;\left|\delta \left(m\right)\right|$$

(4) $$\{\begin{array}{c} R_{u}\;\;,\;if\;\left|\delta \left(m\right)\right|>\bar{\delta \;}/2\;\\ \;R_{d}\;\;,\;if\;\left|\delta \left(m\right)\right|\leq \bar{\delta \;}/2\;\; \end{array}\},\;R=\left\{R_{u}\;,R_{d}\right\}$$

## 3. Results

**Figure 8:**
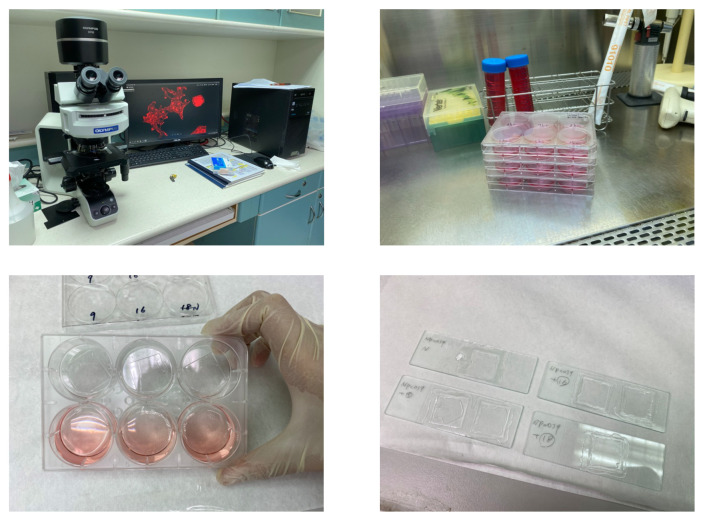
Experimental equipment, cell culture dishes, and cell specimens.

**Figure 9:**
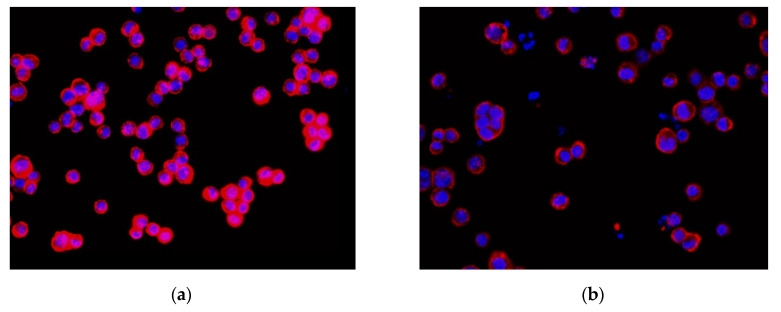
Colon cancer cell images of HCT116: (**a**) HCT116 cell with DMSO(dimethyl sulfoxide) (no.4484); (**b**) HCT116 cell with dinaciclib (no.4496).

In this study, the experimental images were obtained from OLYMPUS D80 digital fluorescence microscopy photographs. The color image resolution was 1360 × 1024 (pixels), and the optical magnification was 40×. Figure 8 shows the equipment used. The image analysis algorithm was programmed using the C++ programming language. Moreover, the YOLO algorithm used in the study was programmed using the C++ programming language. The hardware of the computer included an Intel(R) Core(TM) i7 2.8 GHz Central Processing Unit and an Nvidia RTX 2060 Super Graphics Processing Unit graphics card for cell nuclei image training and executing the proposed method. The cell images included four human colon cancer cell lines (HCT116, DLD-1, HT29, SW480) [39], which were used for studying the treatment regimens and for drug screening. In the experiment, DAPI fluorescent dye was used to stain the cell nuclei and DNA in the cancer cells. Subsequently, rhodamine phalloidin was used to stain the cytoskeleton. Figure 9 shows the HCT116 cancer cell image that was obtained using a fluorescent microscope D80 camera (Figure 9a, untreated cancer cells (image no.4484) with dimethyl sulfoxide (DMSO); Figure 9b, cancer cells treated with dinaciclib (image no.4496)). Notably, dinaciclib (SCH-727965) is an inhibitor of cyclin-dependent kinases, and DMSO is a polar aprotic solvent. In Figure 9, the red color is the cytoskeleton, and the blue color is the cell nucleus. We used the cytoskeleton and cell nucleus images for analysis. As only the cell nuclei that are encapsulated by the cytoskeleton are considered to be intact cells and included in the cell enumeration, the cytoskeleton signal was only used to determine if the cell nucleus could be included for enumeration. The cell nuclei were then the primary analysis targets. The weighting data obtained from training were next used for cell nucleus recognition, while the image processing algorithm and CLSA were used to detect the cell nuclei with mitotic defects and the micronuclei. Experiments were then conducted to validate the performance of this method. The algorithm-related parameters and experimental results are described below.

### 3.1. Nucleus Recognition Results with the YOLO Algorithm

**Figure 10:**
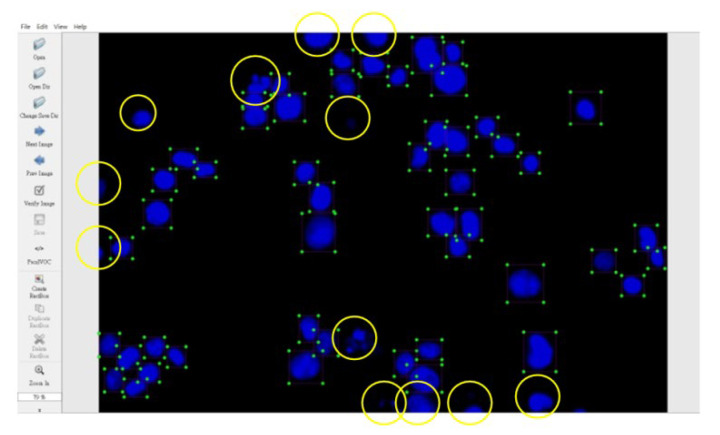
Region selection of the normal cell nuclei for rectangular box annotation using LabelImg (the yellow circles represent abnormal cell nuclei that were not annotated).

**Figure 11:**
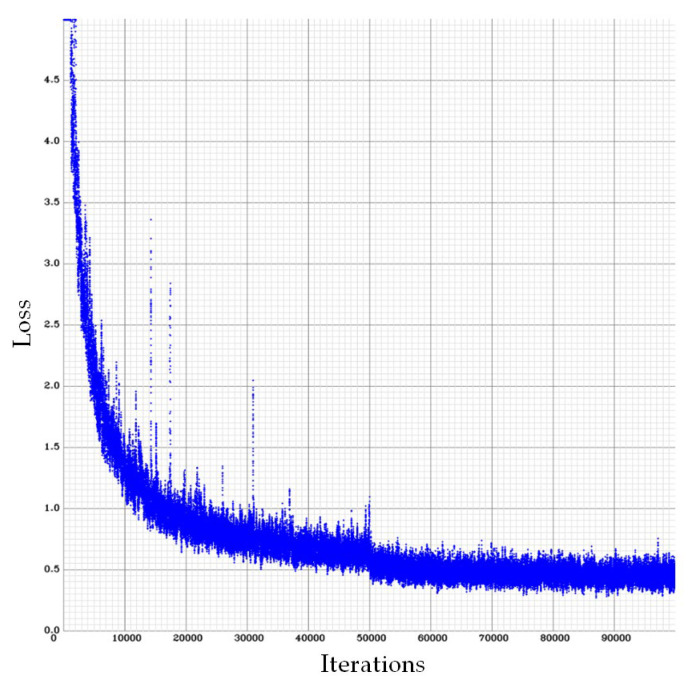
A chart of the loss function in the cell nucleus images trained by the YOLO algorithm (the number of iterations was 100,000, and the average loss was 0.3928).

**Figure 12:**
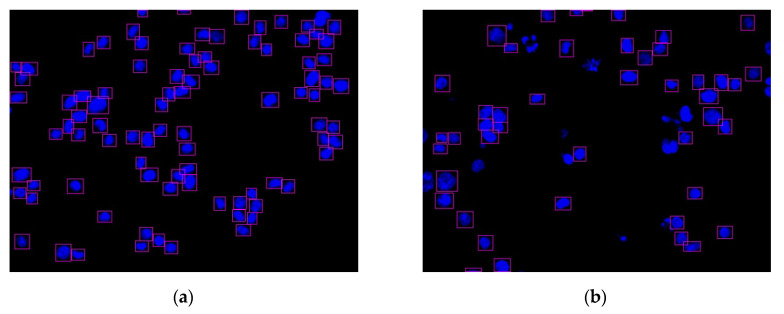
Two nucleus images recognized by the YOLO algorithm: (**a**) HCT116 cell with DMSO (no.4484); (**b**) HCT116 cell with dinaciclib (no.4496).

Consequently, the cell nuclei were the primary analysis targets. The samples of normal cell nuclei were trained only via the proposed method. We collected 92 images for training the neural network (23 images for the model parameter verification and 300 images as the testing data for the four colon cancer cell lines) and labeled the normal cell images using a tool called LabelImg [40]. There were approximately 50 cell nuclei samples in each image. The ratio of the number of training and verification images was 80%: 20%. The region selection of the normal cell nucleus for rectangular box annotation was performed according to the literature [12] and our practical experience with inspection (see Figure 10). In Figure 10, each nucleus in a rectangular box (a purple box with four green points) represents a cell nucleus sample. The abnormal cell nuclei are not annotated in Figure 10 (the yellow circles represent the abnormal cell nuclei). The YOLO algorithm was used to perform 100,000 iterations of training for the normal cell nucleus images. A chart of the loss function in training by the YOLO algorithm is shown in Figure 11 (the average loss was 0.3928). We separated the images of the cytoskeleton and the cell nuclei by extracting the red and blue elements. The same method was also used to recognize the cell nuclei specifically. For this application, the nucleus was the only recognized object. The rectangular box was then used to mark the cell nucleus. Figure 12 (for HCT116 cell) shows the results. The parameters of the YOLO algorithm were the following: The number of training samples was set to 24, and the number of segments to be trained was set to 8. The gradient descent with momentum was set as 0.9, and the weight–decay ratio was set as 0.0005. The learning rate was set as 0.001, and the activation function was Leaky ReLU. The learning policy was “Step”.

### 3.2. Regions without Cytoskeleton Removal and Regions with Incomplete Nucleus Images in the Boundary

**Figure 13:**
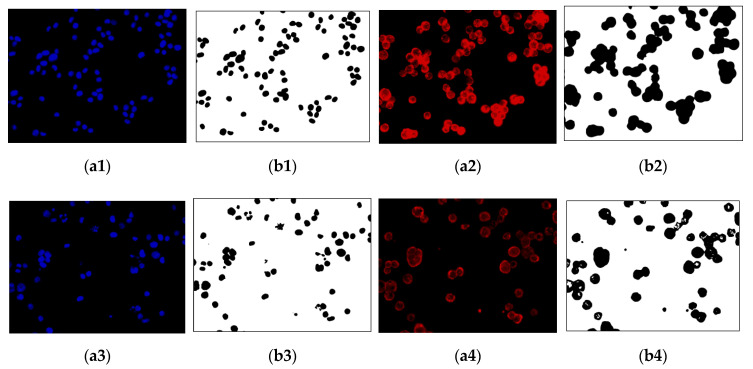
Image color quantization and background removal: (**a1**,**a3**) color quantization of the nucleus images; (**a2**,**a4**) color quantization of the cytoskeleton images; (**b1**,**b3**) nucleus regions (***N***); (**b2**,**b4**) cytoskeleton regions (***C***).

**Figure 14:**
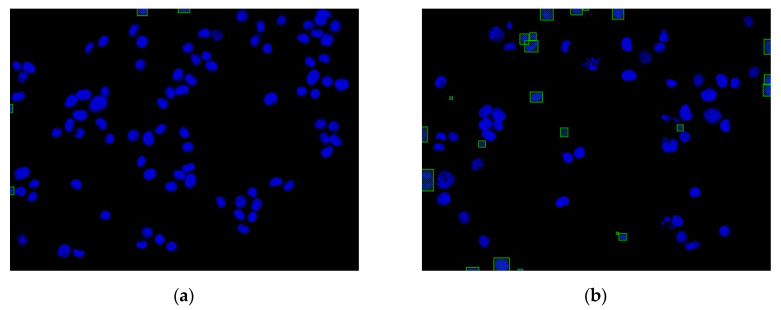
Invalid nucleus regions and regions in boundary detection by ***P*** (=***N***-***B***-***D***) operation (rectangular box with diagonal lines): (**a**) HCT116 cell with DMSO (no.4484); (**b**) HCT116 cell with dinaciclib (no.4496).

Figure 13 shows the function of each step in this experiment. The images of the cell nuclei and cytoskeleton are shown in Figure 13, which is separated from Figure 9. We used k-means clustering [38] for the color quantization of blue elements in the images (the number of quantized colors, k = 15). The background was then removed to obtain the cell nucleus regions, and the lowest quantized color grayscale value was used as the background color. The regions with values higher than the background color are considered to be the cell nucleus and cytoskeleton regions. Figure 13 shows the results: Figure 13a1–a4 show the images after color quantization, while Figure 13b1–b4 show the cell nucleus and cytoskeleton regions after the background is removed. We used the cytoskeleton region (***N***) to obtain the valid cell nucleus regions (***N***∩***C***), and the results are shown in Figure 14. After the ***P*** (=***N−B***−***D***) operation, no valid regions were present in Figure 14a (the four regions are located at the edges of the image). However, Figure 14b contains 10 invalid regions, and 12 regions are located at the edges of the image (rectangular box with diagonal lines).

### 3.3. Color Layer Signature Analysis (CLSA) Algorithm 

**Figure 15:**
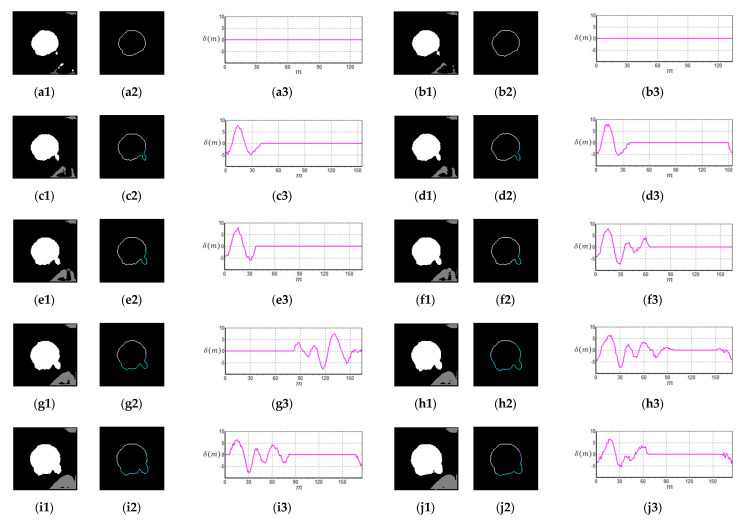
Micronucleus detection of Figure 4a (k = 15, 15th layer is background): (**a1**–**j1**) cell nuclei regions (3rd–12th color layer regions); (**a2**–**j2**) contours of regions; (**a3**–**j3**) short window energy curve according to screening sensitivity (μ = 4).

As the micronucleus is a marker of chromosome instability in cancer cells, the novel CLSA algorithm was designed in this study to detect cell nuclei with micronuclei. In the CLSA algorithm, the k layer region (***A*** = ***Y***∩***C***) in every cell nucleus is used for micronucleus detection. For example, we observed the CLSA test results in Figure 4a, and the results are shown in Figure 15. In Figure 15 (the grayscale value for the first layer is the highest, and the grayscale value for the 15th layer, which is the background layer, is the lowest), the micronuclei appear in the 3rd–12th layers (Figure 15a–j). Here, a1–j1 are the cell nuclei regions, a2–j2 are the cell nuclei contours, and a3–j3 are the short window energy curves according to screening sensitivity. We similarly used k = 15 quantized colors for the experiment, and the short window energy curves of the cell nuclei were used as the test target. Moreover, we did not analyze the color layers that were connected to the edges. To observe changes in the short window energy curve in more layers, we used ten color layers (from the third layer to the twelfth layer). In the results, the micronuclei mostly appear in the color layers with lower grayscale values, as well as in the continuous layers. Therefore, we used a short window energy curve exceeding a μ-range of sensitivity and appearance in three continuous color layers as criteria for the presence of a micronucleus. In the cell nuclei without micronuclei, the texture appeared in the first four layers, while lower layers were not affected by texture. Therefore, to decrease the total number of calculations and avoid the effects of the cell nucleus texture in the first few layers, the middle layers were analyzed. Using k = 15 as an example, the middle fifth layers were used for the short window energy calculations (such as the 4th–12th layers). However, the first fifth layers (the first to third layers) and the last fifth layers (the 13th–15th layers, or the cell nucleus regions connected to the edge regions) were not used for calculations.

### 3.4. The Ratio of the Normal Cell Nuclei to Abnormal Cell Nuclei (Cell Nuclei with Mitotic Defects and Cell Nuclei with Micronuclei) 

**Figure 16:**
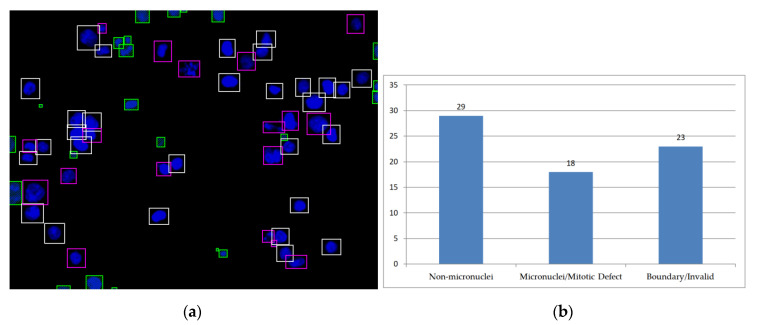
Experiment 1 of the HCT116 cell with dinaciclib (no.4496): (**a**) normal nuclei are distinguished from abnormal nuclei by rectangular boxes; (**b**) histogram of the normal nuclei (non-micronuclei) and the abnormal nuclei (micronuclei/mitotic defects).

Three experiments (k = 15, μ = 4) were conducted on samples from four colon cancer cell lines (HCT116, DLD-1, HT29, SW480). In experiment 1, images from the HCT116 cells treated with dinaciclib were used. In experiment 2, we used an example (a HT29 cell image) obtained from the CellProfiler website [41] to validate our proposed method. In experiment 3, ten image samples (20 images per group) were randomly extracted from every cell line to count the cell nuclei and the ratio of normal cell nuclei to abnormal cell nuclei (cell nuclei with mitotic defects/micronucleus). This process was performed to determine if the proposed method and the manual detection results have similar trends. In experiment 1, image detection was performed on HCT116 cells (Figure 16). The experimental results presented 29 normal cell nuclei (white rectangular box), 18 abnormal cell nuclei with mitotic defects or micronuclei (fuchsia rectangular box), and 23 cell nuclei that were invalid regions (a green rectangular box, not used for ratio calculation). The ratio of normal cell nuclei to abnormal cell nuclei was 61.7% and 38.3%, respectively. Table 1 shows the number of cell nuclei determined by the proposed automatic detection method for cancer cell nucleus images. We also verified the accuracy of the proposed method via the results of manual identification. In Table 1, True Negative (TN) is the number of correctly identified non-micronuclei cell nuclei. False Negative (FN) is the number of incorrectly identified non-micronuclei cell nuclei. True Positive (TP) is the number of correctly identified cell nuclei with micronuclei/mitotic defects/that are in boundary or invalid. False Positive (FP) is the number of incorrectly identified cell nuclei. The values of TN, FN, TP, and FP are taken from the average value of the identification results of the three inspectors. 

**Figure 17:**
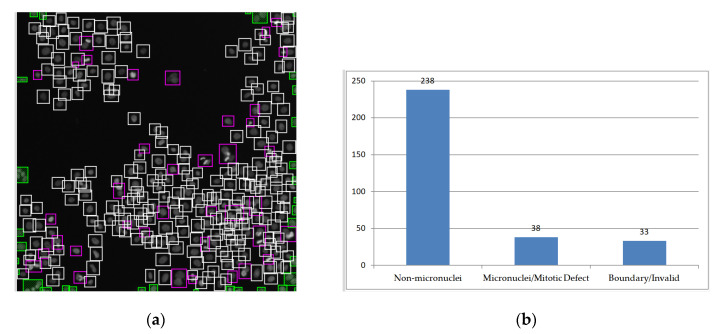
Experiment 2 on the HT29 cell image obtained from the web site of CellProfiler [41]: (**a**) the normal nuclei are distinguished from abnormal nuclei with rectangular boxes; (**b**) histogram of normal nuclei (non-micronuclei) and abnormal nuclei (micronuclei/mitotic defects).

In experiment 2 (Figure 17), detection was performed on the image of the HT29 cells obtained from the CellProfiler website [41]. From the experimental results in Figure 17, there were 238 normal cell nuclei and 38 cell nuclei with mitotic defects or micronuclei (with ratios of 86.2% and 13.8%, respectively) (Table 2 shows the number of cell nuclei). The number of cell nuclei in experiment 2 is shown in Table 2. The number of TN and TP in the two tables is high, which means that the method has a high accuracy and low recognition error. Some cases of micronucleus identification errors in the experiment also remained ambiguous after human judgment.

Finally, experiment 3 examined all images from the four cell lines, and ten images were randomly selected from each cell line for data accumulation. The ratios of the normal cell nuclei and abnormal cell nuclei were calculated, and the results are shown in Table 3. Table 3 compares the manual detection results with the results from the computer vision detection method proposed in this study for the four colon cancer cell lines. Based on the proportional trends of normal cell nuclei and abnormal cell nuclei shown in Table 3, dinaciclib can increase chromosomal instability in cancer cells, resulting in a higher ratio of cells with mitotic defects and micronuclei. This proportional trend is consistent with that observed in the manual detection results and the results from previous studies. The average computation time of the proposed method for an image was less than 10 s. However, it takes at least 5 min to manually complete the detection of one image. For counting the number of nuclei, the difference between the proposed automatic detection method and manual detection is within 10 (among the eight sets of experiments). On average, each image differs by one nucleus. This result verifies that the counting accuracy of the proposed method is close to that of manual counting.

## 4. Discussion and Conclusions

To replace the manual detection of chromosome instability markers in cell nuclei, increase cell detection efficiency and accuracy, and reduce human errors, we proposed an automatic detection method for colon cancer cell nucleus images based on deep-learning analysis and a color layer signature analysis algorithm. More precisely, we proposed a novel CLSA algorithm to detect cell nuclei with micronuclei. During the normal cell nucleus sample selection, we were unable to identify all cell nuclei with micronuclei precisely and could only use cells with clear micronuclei and mitotic defects for selection. In addition to detecting cell nuclei with nonapparent micronuclei, the CLSA algorithm can be used to feedback the results to the cell nucleus samples without micronuclei for selection modification, thereby increasing the accuracy of CNN training. The greater the number of feedback loops, the more accurate the training and test results for cells without micronuclei. However, the CLSA algorithm can only detect a single independent cell nucleus. When two cell nucleus regions are connected, CLSA cannot be used for detection, and the CNN recognition results must be used. Moreover, multiple experiments were conducted in which the YOLO algorithm was used for cell nucleus localization, image preprocessing was used for background removal, invalid regions and regions at the edges of the images were labeled, CLSA was used to detect cell nuclei, and comparative experiments were performed between images of untreated and treated cells. When counting the accuracy of the nuclei, the difference between the proposed automatic detection method and manual detection was within 10. On average, each image differed by one nucleus. Moreover, the trends obtained from the experimental results are similar to those obtained from manual detection, thus indicating greater accuracy in low brightness and nonapparent micronuclei detection. This behavior confirms that the method proposed by this study is feasible.

## Figures and Tables

**Table 1:** The number of cell nuclei (experiment 1).

Total Regions: 70							
Nucleus Detection by YOLO: 45						Free Regions: 25	
Regions within the Image: 38				Regions in the Boundary: 7		Free Regions: 25	
Non-Micronuclei: 29		Micronuclei: 9		In Boundary/Invalid Regions: 23		Regions of Mitotic Defects or Micronuclei: 9	
TN^1^	FN^2^	TP^3^	FP^4^	TP	FP	TP	FP
26	3	8	1	23	0	9	0

TN^1^ is True Negative. FN^2^ is False Negative. TP^3^ is True Positive. FP^4^ is False Positive.

**Table 2:** The number of cell nuclei (experiment 2).

Total Regions: 309							
Nucleus Detection by YOLO: 246						Free Regions: 63	
Regions within Image: 238				Regions in Boundary: 8		Free Regions: 63	
Non-Micronuclei: 237		Micronuclei: 1		In Boundary/Invalid Regions: 33		Regions of Mitotic Defects or Micronuclei: 38	
TN^1^	FN^2^	TP^3^	FP^4^	TP	FP	TP	FP
234	3	1	0	33	0	38	0

TN^1^ is True Negative. FN^2^ is False Negative. TP^3^ is True Positive. FP^4^ is False Positive.

**Table 3:** The ratios between manual detection and the proposed method.

Manual Detection: (The Average Inspection Time Was 5.1 min for One Image) Normal Nucleus: Abnormal Nucleus		The Proposed Method: (The Average Computation Time Was 9.7 s for One Image) Normal Nucleus: Abnormal Nucleus	
HCT116 + DMSO	486: 42 (92.0%: 8.0%)	HCT116 + DMSO	489: 44 (91.7%: 8.3%)
HCT116 + Dinaciclib	335: 113 (74.8%: 25.2%)	HCT116 + Dinaciclib	317: 134 (70.3%: 29.7%)
DLD-1 + DMSO	597: 73 (89.1%: 10.9%)	DLD-1 + DMSO	593: 80 (88.1%: 11.9%)
DLD-1 + Dinaciclib	450: 143 (75.9%: 24.1%)	DLD-1 + Dinaciclib	422: 163 (72.1%: 27.9%)
HT29 + DMSO	546: 54 (91.0%: 9.0%)	HT29 + DMSO	536: 74 (87.9%: 12.1%)
HT29 + Dinaciclib	479: 99 (82.9%: 17.1%)	HT29 + Dinaciclib	473: 106 (81.7%: 18.3%)
SW480 + DMSO	486: 56 (89.7%: 10.3%)	SW480 + DMSO	490: 42 (92.1%: 7.9%)
SW480 + Dinaciclib	615: 125 (83.1%: 16.9%)	SW480 + Dinaciclib	612: 136 (81.8%: 18.2%)
